# Post-spinal Anesthesia Low Back Pain in Obese Female Patients: Comparison of the Median Versus Paramedian Approach

**DOI:** 10.7759/cureus.56784

**Published:** 2024-03-23

**Authors:** Neel Kamal Mishra, Robin Singh, Ravi Prakash, Shefali Gautam, Zia Arshad, Kirtika Yadav

**Affiliations:** 1 Anesthesiology, King George's Medical University, Lucknow, IND; 2 Anesthesiology and Pain Management, Era's Lucknow Medical College and Hospital, Lucknow, IND

**Keywords:** median, comparison, paramedian, obese, low back pain

## Abstract

Background: A side effect of spinal anesthesia is post-dural puncture backache (PDPB), which is characterized by ongoing discomfort at the location of the spinal puncture without any radicular pain. This study aims to compare the incidence and severity of post-dural puncture back pain following spinal anesthesia by median versus paramedian technique in obese female patients.

Methods: A prospective randomized comparative study on 120 female patients, aged 20-50 years with a BMI of 30-40 kg/m^2^ and American Society of Anesthesiologists physical status II, scheduled for elective surgery under spinal anesthesia, was included in the study. Patients were randomly divided into two groups, with 60 patients in each group. Group P uses the paramedian approach for spinal anesthesia, and group M uses the midline approach for spinal anesthesia.

Results: Low back pain incidence was lower in group P than in group M at seven days, but at one month and after, its incidence remained the same in both groups. No difference in the severity of pain was observed.

Conclusions: The occurrence of back pain in the first seven days of surgery was significantly more frequent with the median approach. The pain severity decreased as the time passed from day seven to three months of follow-up. There is no difference in the severity of pain with either approach at different intervals.

## Introduction

Spinal anesthesia (SA) is a common anesthetic technique for lower abdomen and lower limb surgeries. A typical side effect of spinal anesthesia is post-dural puncture backache (PDPB), which is characterized by ongoing discomfort at the location of the spinal puncture without any radicular pain [1]. Incidence of PDPB has been documented from 2% to 29% [2-4]. Pathophysiological reasons for PDPB have been postulated to include regional tissue trauma and/or excessive stretching of spinal ligaments caused by paraspinal muscle relaxation [1]. Post-operative low back discomfort is frequently listed as a typical symptom following SA, regardless of approach. About 9-10% of the study subjects in a Chicago, USA, study who underwent SA experienced post-operative back pain [5]. On the other hand, back pain was the most common postlumbar puncture complaint in multicenter prospective research conducted in Europe, with a 17% occurrence rate [6]. Back pain following spinal anesthesia had a high occurrence rate of 40%, according to prospective observational research of 112 patients in Germany [7].

The median and paramedian approaches, which are both often employed in spinal anesthesia, each have benefits and drawbacks. The median approach is the most frequently utilized technique, but it is technically challenging, particularly in older patients who have structural abnormalities in their spine due to degenerative conditions [4]. The supraspinous ligament, interspinous ligament, ligamentum flavum, and epidural space are all pierced by the needle when it is done by the midline approach. The needle entry point in the paramedian approach was 1 cm lateral and 1 cm caudal to the caudal edge of the spinous process. In this method, the ligamentum flavum is the first structure the needle contacts, and neither the supraspinous nor the interspinous ligaments are pierced [4]. The median approach technique may exacerbate straining of the spinal ligaments, increasing the likelihood of back pain following a spinal puncture. There hasn't been a focused study on how the approach affects back pain after a spinal puncture. The purpose of this study was to compare the incidence and severity of post-spinal anesthesia back pain between median and paramedian techniques in obese female patients.

## Materials and methods

This randomized, open-label, prospective comparative study was conducted over a period of one year. Ethical clearance was obtained from the Institutional Ethical Committee (VII-PGTSC-IIA/P22 dated 21/02/2022) and CTRI registration was done (CTRI/2022/08/044712). A total of 120 female patients aged 20-50 years with a BMI of 30-40 kg/m^2^ and American Society of Anesthesiologists physical status classification II, scheduled for elective lower abdominal, lower limb, and perineal surgeries under spinal anesthesia, were included in the study. Patients undergoing emergency surgery, pre-existing low back pain, on analgesic medication, more than two punctures attempted, previous spinal/epidural anesthesia, previous spine surgery, and pregnancy were excluded from the study. Informed and written consent was obtained from patients.

The sample size was calculated on the basis of the incidence of newly developed post-operative low back pain [4] in two study groups using the formula

n = (zα + zβ)2/[1n(1−e)]2÷[(1−p1/p1)+(1−p2/p2)],

where p1 = 0.36 (36%) incidence of newly developed post-operative low back pain in the first group, p2 = 0.16 (16%) incidence of newly developed post-operative low back pain in the second group, e = 1.25 (p2/p1), the risk ratio considered to be clinically significant, type I error, α = 5%; type II error, β = 20% for setting the power of study 80%. The sample size is calculated to be n = 60.

A preoperative preliminary assessment was performed on all patients, and randomization was done using a computer-generated random number table. Patients were divided into two groups, with 60 patients in each group: Group P (paramedian group), where the paramedian approach for spinal anesthesia was used. Group M (median group), where the midline approach for spinal anesthesia was used.

In the operating room, standard American Society of Anesthesiologists (ASA) monitoring was started. With proper aseptic precautions, spinal anesthesia was performed with 0.5% hyperbaric bupivacaine at the level of L3-L4 or L4-L5 intervertebral space. On day seven, one month, two months, and three months, the severity and incidence of back pain were noted. The incidence and severity of back pain were noted. The severity of pain was assessed using a 10-point VAS. <4 is mild, 4-6 is moderate, and >6 is severe pain. All spinal anesthesia was performed in a sitting position by the same anesthetist using a 25G Whitacre needle (Becton and Dickinson Company, Allschwil, Switzerland). Each skin puncture was considered as one attempt. The patient was assessed for low back pain in the post-operative period. Statistical analysis was done using IBM SPSS Statistics for Windows, Version 25 (Released 2011; IBM Corp., Armonk, New York, United States). The values were expressed as the mean with a standard deviation. Student’s t-test and ANOVA were used for statistical analysis.

## Results

A total of 150 obese female patients were enrolled. Out of 150, 20 patients were excluded due to more than two attempts, and 10 patients were lost in follow-up. A total of 120 patients, 60 patients in group M and 60 patients in group P, were assessed and analyzed. 

The mean age was 36.01±9.40 years in group M and 35.00±7.88 years in group P. The mean age was not significantly different between groups. The mean weight (kg), height (cm), and BMI (kg/m^2^) were 76.07±9.64, 152.65±1.71, and 31.92±1.35 in group M and 76.40±3.22, 153.53±2.27, and 31.27±1.10 in group P, respectively. The mean height (cm) and BMI (kg/m^2^) were not significantly different between groups (Table 1).

**Table 1:** Association of mean weight (kg), height (cm), age, and BMI (kg/m2) between group M and group P.

	Group M (n = 60)	Group P (n = 60)	T	p-value
	Mean±SD	Mean±SD		
Weight (kg)	76.07±9.64	76.40±3.22	−0.25	0.800
Height (cm)	152.65±1.71	153.53±2.27	−2.81	0.215
BMI (kg/m^2^)	31.92±1.35	31.27±1.10	2.89	0.411
Age (in years)	36.01±9.40	35.00±7.88	2.01	0.325

The percentage of first and second attempts was 86.67% and 13.33% in group M; 93.33% and 6.67% in group P. Based on the number of attempts, both groups were comparable (Table 2). 

**Table 2:** Comparison of frequencies of no. of attempts between group M and group P.

No. of attempts	Group M (n = 60)		Group P (n = 60)		Chi-sq.	p-value
	n	%	n	%		
1	52	86.67	56	93.33	0.83	0.361
2	8	13.33	4	6.67		

The incidence of pain was 36.67% in group M and 18.33% in group P at seven days. The pain was significantly lower in group P as compared to group M at seven days but not significantly different at one, two, and three months (Table 3). 

**Table 3:** Comparison of incidence of pain between group M and group P. * p<0.05 is significant.

	Group M (n = 60)		Group P (n = 60)		Chi-sq.	p-value
	n	%	n	%		
Pain at seven days	22	36.67	11	18.33	4.18	0.041*
Pain at one month	12	20.00	6	10.00	0.00	0.64
Pain at two months	5	8.33	2	3.33	0.12	0.732
Pain at three months	3	5.00	1	1.67	0.06	0.809

No difference in severity of pain was observed at seven days, one month, two months, and three months (Table 4). 

**Table 4:** Comparison of pain severity between group M and group P.

Post-operative day	Pain severity	Group M		Group P		T-value	p-value
		n	%	n	%		
At seven days	No	38	63.33	49	81.67	6.02	0.111
	Mild	15	25.00	8	13.33		
	Moderate	5	8.33	3	5.00		
	Severe	2	3.33	0	0.00		
At one month	No	48	80.00	54	90.00	3.35	0.187
	Mild	10	16.67	6	10.00		
	Moderate	2	3.33	0	0.00		
	Severe	0	0.00	0	0.00		
At two months	No	55	91.67	58	96.67	1.37	0.243
	Mild	5	8.33	2	3.33		
	Moderate	0	0.00	0	0.00		
	Severe	0	0.00	0	0.00		
At three months	No	57	95.00	59	98.33	1.03	0.309
	Mild	3	5.00	1	1.67		
	Moderate	0	0.00	0	0.00		
	Severe	0	0.00	0	0.00		

## Discussion

The most common regional anesthesia technique used in many different surgical procedures, such as urogenital surgeries, cesarean sections, and lower limb surgeries, is spinal anesthesia. Although spinal anesthesia is the preferred method, it has more incidence of back pain than general anesthesia [8-11].

In this randomized, prospective study, we compared the incidence and severity of post-dural puncture back pain following the performance of the median and paramedian techniques in obese female patients. The lower incidence of post-dural puncture back pain in the early post-operative period with the paramedian technique may be due to a lesser structure being pierced and less tissue being damaged compared to the median technique. According to Lee et al., the technique of median access was more frequently associated with PDPB seven days after surgery compared to paramedian access. In addition, there was no evidence of a difference in pain intensity between groups throughout the survey period, and PDPB lasting longer than three months was rare regardless of treatment [4]. Rhee et al. reported that back discomfort was cited by 29% of these unsatisfied patients as the cause of their unhappiness. The following are risk factors for PDPB: prior back discomfort, immobilization of the spine for more than 2.5 hours, position of the lithotomy during surgery, body mass index greater than 32 kg/m^2^, and repeated efforts to insert the needle [2]. According to Zeleke et al., the prevalence of PSBP was 11.3% on the second day (95% CI: 6.0, 12.8), 18.1% (95% CI: 8.6, 18.1) on the third day, and 18.1% (95% CI: 8.6, 23.1) on the first day [9]. In studies done in China and Turkey, the incidence of PSBP on the first post-operative day was higher, i.e., 38% and 29.3%, respectively [10]. Back discomfort was reported by 38, 29.9, and 31.6% of patients, respectively, on the first, second, and third post-operative days, according to a study done in Asella, Ethiopia [11].

Injury to the ligaments, fascia, and bone, along with localized inflammation, are the mechanisms that cause PDPB. Potential aggravating factors include spinal immobilization, spinal anesthesia-induced paraspinal muscle relaxation, flattening of the normal lumbar convexity curve, and stretching and/or straining of the facet joints and paraspinal ligaments, particularly when the patient is in the lithotomy position [4]. The symptoms of PDPB include low severity, localized discomfort at the injection site, response to oral inflammatory medications, and spontaneous resolution.

The severity of back pain decreases with the passage of time, with only two patients complaining of pain after three months. According to a recent study, the frequency of PDPB dropped from 29% one day after spinal anesthesia to 5% four weeks later, and the severity of the pain also lessened over time [12]. The results of the current study agreed with those of earlier ones [9-11].

The limitations of our study include a small sample size, and the results of our study are only applicable to obese female patients. It was a single-center study. A multicenter study with a large sample size will provide better results.

## Conclusions

Median and paramedian approaches for spinal anesthesia have similar incidence and severity of low back pain in obese females, except at one week of surgery, where the incidence is lower in the paramedian approach. The pain severity decreased as the time passed from day seven to three months of follow-up. There is no difference in the severity of pain with either approach at different intervals.
